# A novel transcriptome-derived SNPs array for tench (*Tinca tinca* L.)

**DOI:** 10.1371/journal.pone.0213992

**Published:** 2019-03-19

**Authors:** Girish Kumar, Jorge Langa, Iratxe Montes, Darrell Conklin, Martin Kocour, Klaus Kohlmann, Andone Estonba

**Affiliations:** 1 Research Institute of Fish Culture and Hydrobiology, South Bohemian Research Center of Aquaculture and Biodiversity of Hydrocenoses, Faculty of Fisheries and Protection of Waters, University of South Bohemia in Ceske Budejovice, Czech Republic; 2 Department of Genetics, Physical Anthropology and Animal Physiology, University of the Basque Country UPV/EHU, Leioa-Bilbao, Bizkaia, Spain; 3 Department of Computer Science and Artificial Intelligence, University of the Basque Country UPV/EHU, San Sebastian, Gipuzkoa, Spain; 4 IKERBASQUE, Basque Foundation for Science, Bilbao, Bizkaia, Spain; 5 Department of Aquaculture and Ecophysiology, Leibniz-Institute of Freshwater Ecology and Inland Fisheries, Berlin, Germany; National Cheng Kung University, TAIWAN

## Abstract

Tench (*Tinca tinca* L.) has great economic potential due to its high rate of fecundity and long-life span. Population genetic studies based on allozymes, microsatellites, PCR-RFLP and sequence analysis of genes and DNA fragments have revealed the presence of Eastern and Western phylogroups. However, the lack of genomic resources for this species has complicated the development of genetic markers. In this study, the tench transcriptome and genome were sequenced by high-throughput sequencing. A total of 60,414 putative SNPs were identified in the tench transcriptome using a computational pipeline. A set of 96 SNPs was selected for validation and a total of 92 SNPs was validated, resulting in the highest conversion and validation rate for a non-model species obtained to date (95.83%). The validated SNPs were used to genotype 140 individuals belonging to two tench breeds (Tabor and Hungarian), showing low (F_ST_ = 0.0450) but significant (<0.0001) genetic differentiation between the two tench breeds. This implies that set of validated SNPs array can be used to distinguish the tench breeds and that it might be useful for studying a range of associations between DNA sequence and traits of importance. These genomic resources created for the tench will provide insight into population genetics, conservation fish stock management, and aquaculture.

## Introduction

Tench (*Tinca tinca* L.) is a freshwater fish species within the *Cyprinidae* family that spawns and grows ideally at water temperatures of 20–29°C [1, 2]. Its native distribution is Eurasia; however, due to human-mediated movement, tench can also be found in temperate and tropic freshwater regions across the globe [3]. Due to its attractive appearance and specific meat flavour, tench has relevant economic importance and is commonly used in aquaculture and sport fishing [4]. For example, tench farming is a common aquaculture activity in Europe and has recently expanded to China [5]. All of these facts motivate the increase of its annual global aquaculture production [6] of about 1400 tons [7].

Tench has very interesting features that set the species apart from other members of the *Cyprinidae* and that have popularized tench as an experimental model [8]. These include: an unequivocal body colour, normally green to brown-green, with golden, blue and albinotic phenotypes also existing [9]; small and hardly visible scales deeply embedded into the dermis; obvious sexual dimorphism in pelvic fins [4], specific reproductive biology [1]; low incidence of viral and bacterial diseases but high susceptibility to some chemical compounds [10]; and monophyletic origin (all descendants of a common ancestor) within *Tinca* genus [11]. Genetics studies have also shown that tench is still a diploid species (2n = 48) [12], which is advantageous for some genetic studies, compared to many cyprinids that are polyploid species [13].

Genetic studies on tench have until now been based on allozymes [14, 15], microsatellites [16, 17], PCR-RFLP [18, 19] and sequence polymorphism of genes and DNA fragments [20–22]. These studies have revealed the existence of Western and Eastern phylogroups [6, 19]. Individuals from both phylogroups have undergone natural and human-aided hybridization and this has produced hybrids that appear in natural water bodies as well as in cultured stocks along Europe.

The rapid development and application of sequencing technologies is now permitting researchers to discover thousands of SNPs at relatively low cost compared to the traditional Sanger sequencing method [23]. Transcriptome sequencing is considered a cost-effective strategy for discovering SNPs in non-model species. In fact, as a transcriptome is directly associated with functional regions in a genome, transcriptome-derived SNPs can be informative for adaptive variation [24–26] and they can be used not only for assessing population genetic structure, but also for genomic selection for traits of interest to aquaculture such as growth, sex determination or disease resistance (e.g. [27–29]). Given these advantages, SNPs derived from transcriptomes have been widely discovered and studied in many fish species [29–42].

The aim of this study was: to discover and validate transcriptome-derived SNPs in *T*. *tinca*, based on the strategy designed by Montes *et al*. and successfully applied in other fish species [38, 43]. The SNPs array was then used to disentangle the population genetic structure of two cultured tench breeds (Tabor and Hungarian), previously identified as stocks representing mixture of haplotypes out of both phylogroups [22].

## Materials and methods

### Ethics statement

The handling and usage of experimental fish in this study was done in accordance with the Czech Act. No 256/1992 Coll. as amended under supervision of the Institutional Animal Care and Use Committee (IACUC) of the University of South Bohemia (USB), Faculty of Fisheries and Protection of Waters (FFPW) in Vodňany. The USB FFPW has approval of the Ministry of Agriculture of the Czech Republic for handling and usage of experimental animal’s ref. no. 16OZ15759/2013-17214. The presented study was included in the planned activities dealing with study of biodiversity, genetic, physiological and reproductive variability and performance of selected freshwater fish species. The experimental stock was reared under the common semi-intensive pond management conditions. The fish sacrificed for the study were euthanized in accordance with the Ordinance no. 419/2012 Coll. as amended. The fish were euthanized by blow into the head using a blunt object and bleeding. One of the co-authors was present during handling and processing the fish owned the certificate (no. 0135/2000-V3) which allows him to conduct and manage experiments involving animals according to the above mentioned act.

### Sample collection

In the methodology followed for SNP discovery, two samplings (corresponding to the two sequencing approaches) were performed; one for transcriptome sequencing, and another for genome sequencing.

For transcriptome sequencing, 4 tench individuals (2 males and 2 females) were sampled. The sampled individuals belonged to two metabolic activities (summer season with 20°C water temperature, and winter season with 4°C water temperature) and two breeds (Hungarian and Tabor) cultured in Vodňany, Czech Republic since 1990’s [44] (present Faculty of Fisheries and Production of Waters, University of South Bohemia in České Budějovice). The Tabor breed was established by collecting fish from ponds of a Czech county, and the Hungarian breed by introducing the tench from Hungary. To increase the homozygosity, inbreeding and gynogenesis within each breed were applied. Both breeds, containing approximately 120 adult individuals, have been maintained to date by intra-linear mating only for 6 generations. Previous studies on these fish have shown that both breeds have gene pools mixed of both Western and Eastern phylogroups [22, 45]. The transcriptome changes according to genes expressed. Expression of various genes depends on many inner and outer factors (e.g. fish age, health status, phase of reproductive cycle, weather, season—growing or wintering etc.). That is why we sampled fish in winter (no-growing season) and summer (growing season) in order to cover different genes expressed in mature 4-year old fish. Each fish was humanely sacrificed and two different tissues were collected- whole brain (without pituitary) and back muscle (approx. 1 g) and immediately frozen in liquid nitrogen, and stored at -80°C until RNA extraction was performed. We had eight initial tissue samples, though two (brain in both cases) were not suitable for sequencing due to RIN values below 8. The remaining six samples (two of them in duplicate) were used for library construction and Illumina sequencing (S1 Table).

For genome sequencing, a total of ten tench individuals from six different locations were collected (S2 Table) in order to cover maximal available genetic diversity, including phylogroup origin of tench species. Samples were taken from the tench tissue collection of Leibniz Institute of Freshwater Ecology and Inland Fisheries, Berlin, Germany and they represented populations throughout Neighbor-joining trees inferred from studies focused on genetic diversity of the growth hormone (GH) gene [22], microsatellites [17] and mitochondrial DNA [18].

### RNA and DNA extraction

Total RNA was isolated using Qiazol lysis reagent (Qiagen). The isolated RNA was quantified with a Nanodrop 2000 (Thermo Scientific) and integrity of RNA (RIN) was determined using an Agilent 2100 Bioanalyzer (Agilent Technologies). Samples with RIN values above 8 were used for RNA sequencing, and used for library construction and Illumina sequencing. According to the RNA quality standards, six samples were sequenced (S1 Table).

Genomic DNA was isolated from muscle, fin or blood samples using the peqGOLD Tissue DNA Mini Kit (Peqlab Biotechnologie) following manufacturer instructions. The quantity and quality of DNA was measured with Qubit 2.0 Fluorometer and 0.8% agarose gel electrophoresis. The DNA samples with concentrations ≥ 50 ng/μl, 260/280 ratios of 1.8–2.0 and clear high molecular weight bands on the gel were used for genome sequencing. An equimolar amount of total DNA was then pooled for the library preparation.

### Library construction and Illumina sequencing

A multiplex sequencing library was prepared by labeling each sample (six RNA samples, two of them replicated; and two DNA pools) with specific 10-mer barcoding oligonucleotides. Transcriptomic and genomic libraries were sequenced in a single lane of Illumina HiSeq2000 and HiSeq2500 platforms, respectively. Sequencing reactions were performed separately for transcriptome and genome with paired-end 101 bp and 126 bp reads, respectively. Sequencing was performed at CNAG- Centre Nacional d’Anàlisi Genòmica, Barcelona, Spain. All sequence data have been submitted to NCBI’s submission portal under the BioProject accession number PRJNA414567.

### Genome size estimation

We estimated the genome size of *Tinca tinca* by means of the frequencies of the kmers in the DNA reads. Reads were processed with Jellyfish 2.2.10 [46] using the *count* subcommand with a kmer size of 25. The frequencies were computed using the *histo* subcommand. Finally, the genomic haploid length, along with the repetitive and unique contents and rate of heterozygosity, was computed using the GenomeScope web service [47].

### Transcriptome *de novo* assembly and annotation

Raw RNA reads were processed in a strict four-step procedure in order to obtain a high-quality reference. First, adaptors and low-quality reads were removed with Trimmomatic v0.33 [48] by deleting the first 13 nucleotides of the read. Removal of adapters was done with the ILLUMINACLIP:TruSeq3-PE-2.fa:2:30:10 parameters by setting a minimum mean PHRED quality value of 10, trailing bases with quality value at least 20, and a minimum read length of 31 bases. Second, contaminants indicated by the UniVec database were removed with SeqClean (https://sourceforge.net/projects/seqclean/). Third, Trimmomatic was run on Single End mode to remove low quality and excessively short reads with the following parameters: minlen:31 avgqual:10 minlen:31 trailing:19 minlen:31 tophred33. Finally, the paired-end structure of the reads was recovered with a custom script written in Python with help of the Biopython package [49].

After the transcriptome reads were trimmed, paired and unpaired high-quality reads (all RNASeq data) were assembled into contigs using Trinity v2.0.6 [50]. The resulting transcriptome was uploaded to NCBI Transcriptome Shotgun Assembly Sequence Database and it is available at GenBank with accession number GFZX00000000.1. Full implementation of assembly procedure is available at https://github.com/GenomicResources/ttin_assembly.

To measure the quality of the assembled transcriptome, we used a two-fold approach. First we backmapped (with Bowtie2) the trimmed reads against the generated reference to measure the fidelity of the assembly with respect to the reads. According to the authors of Trinity, transcriptomes with mapping rates above 80% are considered good assemblies. Second, we used BUSCO v3.0.2 [51, 52] to assess the quality of the assembly by searching for *Actynopterygii* Single Copy Orthologs (SCOs). The program searches the homology between our transcriptome and a set of precomputed proteins that are known to be conserved across the evolution of a large set of species, classifying them as SCOs, conserved but duplicated, fragmented, or missing.

Finally, TransDecoder v2.0.1 (https://transdecoder.github.io/) and Trinotate (http://trinotate.github.io/) were used for transcriptome annotation and generation of a tench proteome. Transdecoder is a pipeline that extracts the possible open reading frames (ORFs) from a raw transcriptome to predict if it has homology with BLAST [53] against a protein reference database like Swiss-Prot [54] (downloaded on August 2015), UniRef90 [55] (accessed on August 2015), or homology via Hidden Markov Models with HMMER [56] (retrieved on August 2015) by querying the Protein Families database (Pfam, [57]. Once ORFs are called and possible homologies to elements in the different databases are hypothesized, a proteome is generated.

The next step in the procedure is the annotation of both the transcriptome and the predicted proteome developed as described above with Trinotate. It consists of homology searches, as done in the TransDecoder step, with help of BLASTX, BLASTP and HMMER, to then make use of a database (downloaded on August 2015) containing annotations from Gene Ontology (The Gene Ontology Consortium, 2000), KEGG [58], and eggnog [59].

Chimeras and duplicated regions were filtered out from the assembled transcriptome with stringent filters. First, contigs were quantified with Kallisto [60] and those with zero counts were removed with help of the Sleuth R package [61]. Additionally, according to the generated proteome, contigs with no coding potential were removed. Finally, genes that produce two or more isoforms were deleted. These procedures were performed using custom scripts in Python, R (R Core Team 2016), SAMtools [62] and Snakemake [63]. Implementation of the annotation procedure is available online at https://github.com/GenomicResources/ttin_trinotate. The resulting filtered transcriptome was used in the following steps of intron-exon boundary (IEB) prediction and SNP discovery.

### SNP calling and IEB prediction

Tench SNP calling was performed as described by [38]. Two parallel SNP calling approaches were performed by aligning transcriptome (T2T) and genome (G2T) trimmed reads to the filtered transcriptome. This alignment was performed with Bowtie2 in local mode [64]. In this pipeline, PCR duplicates from both transcriptome and genome reads were removed using the SAMtools *rmdup* command [62]. Subsequently, variants were called with SAMtools *mpileup* command [62]. In order to avoid false SNPs, a maximum contig depth of 200x was set to avoid both repetitive sequences and false positive local alignments; the minimum contig depth allowed for T2T variants was 8x and 20x in the case of G2T variants in order to remove transcripts with low coverage that could bias the SNP calling procedure; the minimum variant count allowed for T2T variants was 2 high quality (HQ) bases (i.e., the alternative base appears at least twice), and 3 HQ bases for G2T variants. This last filtering step requires the SNPs to have higher MAFs when coverage is lower. After applying all of these filters, only common variants present in both T2T and G2T SNP discovery approaches were considered as putative SNPs. The implementation of the transcriptome filtering and SNP calling procedures is available online at https://github.com/GenomicResources/ttin_snps.

It is well known that genotyping procedures (for PCR based technology like fluidigm) will fail if primers are spanning or otherwise close to intron-exon boundaries [65]. Therefore, the filtered transcriptome reference was *in silico* assessed to detect IEBs as described by [66]. This is done by mapping genomic reads to the transcriptome, and computing p-values for *change points*. These are locations in the transcriptome where one or more genomic reads do not map throughout their whole length but rather the mapping is initiated or terminated internally to the read. Locations with low p-values represent a surprising number of change points at that location, hence a likely IEB. Predicted IEBs are annotated and avoided during genotyping primer design.

### SNP genotyping and validation

A total of 140 tench samples belonging to two breeds (Tabor, N = 66 and Hungarian, N = 74) were genotyped for selected subset of 96 candidate SNPs. Only one SNP per contig was chosen and selection was not biased to any gene family. As growth-related traits are of main importance in most cultured fish species and growth hormone (GH) gene might be associated with growth [6], the SNP array was within each breed also associated with GH gene genotype distinguishing alleles of Eastern or Western phylogroup haplotype. Assignment of an individual to Eastern (E) only, Western (W) only or hybrid (H) GH gene genotype was performed using the sequence analysis of GH gene [22]. In the pure Western GH gene genotype the first GH gene fragment including polymorphic side 1 (PS1) and the second GH gene fragment including PS7 were 344 bp and 451 bp long, respectively, while the individuals of pure Eastern GH gene genotype had fragments of 341 bp and 455 bp in length, respectively. In hybrids, haplotypes of both phylogroups were observed (i.e. 341 and 344 for PS1 and 451 and 455 for PS7). Flanking sequences of a subset of SNPs selected for validation were used for primers and probe design according to Fluidigm Genotyping System requirements. After genotyping, SNPs were categorized as *no signal* (unamplified SNPs), *disperse* (call rate < 80%), *monomorphic* (minor allele frequency, MAF < 0.01) and *psv* (paralogous sequence variant; all individuals are heterozygotes). For the conversion rate (proportion of all genotyped SNPs showing polymorphism), *no signal* and *disperse* SNPs were discarded, while only polymorphic SNPs (no *monomorphic*, neither *psv*) were used for the estimating the validation rate (proportion of polymorphic SNPs reliably scored in a sample of individuals). Polymorphic SNPs were uploaded to EBI’s European Variation Archive under the study accession number PRJEB23783.

### Population genetic structure

For each polymorphic SNP, minor allele frequency, and expected and observed heterozygosities (*H*_*e*_ and *H*_*o*_, respectively) were estimated using the software package GeneClass2 [67]. Deviations from Hardy-Weinberg equilibrium (HWE) were evaluated for each *locus* using Fisher’s exact test implemented in Genepop 4.0 [68] with 10,000 dememorizations, 100 batches and 5,000 iterations per batch.

To determine the genetic structure of tench individuals, genotype data were analyzed with Structure 2.3.4 software [69]. The number of clusters *k* was determined by comparing log-likelihood ratios in 10 runs for values of *k* between 1 and 10. Each run started with a burn-in period of 10,000 steps followed by 100,000 MCMC replicates. The optimal *k* was estimated as proposed by [69] and [70] and bar plots were generated using Pophelper v1.0.7 [71].

Based on this initial structure, the Bayesian likelihood method implemented in Bayescan 2.1 [72] was used to detect loci under natural selection (outlier loci). Bayescan was run with twenty pilot runs of 5,000 iterations, an additional burn-in of 50,000 iterations and prior odds of 10 for neutral model. Critical values were adjusted with a false discovery rate (FDR) procedure (q<0.1) [73]. Results of the outlier test were used to partition the SNP dataset into neutral and outlier loci; i.e., markers presumably under natural selection. Those loci resulting as outlier were removed from prospective analysis, regarding neutral variation, and annotations of the genomic regions including those loci were re-inspected.

Finally, neutral genetic differentiation and inbreeding were assessed. Neutral genetic differentiation was estimated with unbiased F_ST_ (distance matrix: pairwise difference) [74] using Arlequin v3.5 [75]. Inbreeding was estimated with F_IS_ [74] statistic using Fstat software [76]. The statistical significance of F_ST_ and F_IS_ was tested by 1,000 permutations for each pairwise comparison. In all cases with multiple comparisons, error rates were corrected using the sequential Bonferroni procedure [77].

## Results

### Transcriptome and genome sequencing

In total 32 million paired-end transcriptomic reads, with an average length of 101 bp, were sequenced (S3 Table). In the case of genome, 316 million genomic reads with a read length of 126 bp were generated, encompassing 154 million reads generated for Western pool (19.6 Gbp), and 162 million reads for Eastern pool (20.4 Gbp). GenomeScope estimated that the *Tinca tinca* has a maximum genome size of 778,555,248 base pairs, where 599,234,146 base pairs (76.97%) constitute unique regions (S4 Table; S1 Fig). Overall, genome sequences constituted an estimated 51.58x coverage of the tench genome.

### Transcriptome *de novo* assembly and annotation

Trimming of raw transcriptome reads did not result in a significant removal of reads, but 16% of nucleotides were discarded (S5 Table). The transcriptome *de novo* assembly consisted of 267,058 contigs (294.7 Mbp), which are the result of potentially 174,378 genes. The length of the assembled contigs ranged from 224 bp to 23,703 bp with an average length of 1,103 bp (S2 Fig).

Given the high number of sequences that Trinity yielded, we assessed the quality of our transcriptome by read mapping and by the contents of Single Copy Orthologs. On the one hand, the backmapping method achieved mapping success rates between 96.54% and 99.38% (S6 Table), suggesting therefore a good reconstruction of the *Tinca tinca* transcriptome. On the other hand, BUSCO reported that the transcriptome contains 85.9% of the Actinopterygii BUSCOs (where 40.4% are single copies), 6.7% are fragmented, and only 7.4% are completely missing (S7 Table). We conclude that given that even though we only sampled two tissues (muscle and brain) of *Tinca tinca*, this assembly is a good representation of the transcriptome.

According to the gene-isoform distribution in S3 Fig the distribution is skewed towards genes composed by one transcript. There are 10,705 genes of that composition (out of 174,378 genes, 86.42%, and out of 267,058 isoforms, 56.43%). The mean of the distribution is 1.53 transcripts per gene. As an extreme value, there is a gene (possibly a gene family) composed of 55 transcripts.

Regarding annotation, 89,832 transcripts were annotated (33.63%) as 126,187 proteins and 32,619 genes. From these, 64,676 transcripts (105,812 proteins and 9,295 genes) had a positive match to the UniRef90 database with *blastp* (S8 Table); similarly, 101,606 contigs (39,169 genes) were positively mapped with *blastx* (S9 Table). In both cases, top reference transcripts belonged to the same species: *Danio rerio*, *Astyanax mexicanus*, *Oncorhynchus mykiss*, *Oreochromis niloticus*, and *Ictalurus punctatus* (S4 and S5 Figs; S10 Table).

Overall, 67,953 contigs (77,626 proteins and 22,996 genes) were positively matched to 5,054 different protein domains according to the Pfam database (S6 Fig). The five most popular domains were: C2H2-type zinc finger (6.19%), Immunoglobulin domain (4.02%), Ankyrin repeat (3.22%), Leucine rich repeat (3.06%), and Zinc finger, C2H2 type (2.58%; S11 and S12 Tables).

According to the EggNOG database, 43,291 contigs (43,366 proteins and 14,714 genes) had a match against 3,338 different elements of the EggNOG database, including Serine Threonine protein kinase (7.63%), repeat-containing protein (3.03%), Zinc finger protein (2.95%), Ankyrin repeat (2.47%) and GTP-binding protein (1.27%) (S7 Fig and S13 Table).

Finally, Gene Ontology (GO) analysis showed 88,031 contigs (89,014 proteins and 30,345 genes). The highest number of GO terms was assigned to biological processes (48.63%) followed by molecular functions (29.66%) while cellular component has the least assigned terms (21.70%; S8 Fig). The three most commonly assigned GO terms in biological process category were genes involved in *Transcription*, *DNA-templated* (2.03%), *Regulation of Transcription*, *DNA-templated* (1.38%) and *Signal Transduction* (0.73%). In the molecular function ontology, *ATP binding* (5.77%), *Metal ion binding* (5.32%), *Zinc ion binding* (4.08%) and *DNA binding* (4.06%) were the most represented terms. The three major assigned GO terms for cellular component were nucleus (10.51%), cytoplasm (10.35%) and integral components of the membrane (7.26%; S9–S12 Figs; S14 and S15 Tables).

### SNP discovery and validation

According to kallisto, a total of 262,801 contigs (out of 267,058) had an expression value above zero transcripts per million (TPM). Therefore 98.41% of the original assembly remained valid for further analysis. From those, 89,832 contigs were identified as having no coding potential and were discarded. Finally, contigs representing more than one isoform were also removed. After all these filters, the transcriptome was reduced to 18,479 contigs spanning 20.32 Mbp.

The filtered transcriptome was used as reference for mapping genome (G2T) and transcriptome (T2T) trimmed reads. The trimming process did not significantly decrease the number of transcriptome or genome reads (S16 and S17 Tables). The mapping process resulted in 19.51% of genomic reads and 22.63% of transcriptome reads assigned to the filtered transcriptome (S18 Table). From these mappings, a total of 131,188 G2T SNPs were called in 15,593 transcripts (8.41 G2T SNPs/transcript; Table 1), and 98,869 T2T SNPs were called in 13,721 transcripts (7.21 T2T SNPs/transcript). Together, G2T and T2T called 169,643 SNPs in 16,263 transcripts, but only 60,414 SNPs in 11,769 transcripts (5.13 SNPs/transcript) were common to both sets. These 60,414 SNPs represented the final set of putative SNPs discovered in the tench transcriptome.

**Table 1:** Descriptive statistics of G2T, T2T and common discovered SNPs.

	G2T	T2T	Common
**Contigs with SNPs**	15,593	13,721	16,263
**Number of contigs in filtered assembly**	18,479	18,479	18,479
**Transcripts with SNPs (%)**	84.38	74.25	88.01
**SNPs number**	131,188	98,869	169,643
**Assembly size (bp)**	20,316,163	20,316,163	20,316,164
**Mean mutation rate (SNPs/bp)**	0.006	0.005	0.008
**SNPs per transcript**	8.41	0.14	0.10

Regarding IEB avoidance, 4,091 transcripts out of 18,479 were signaled as not having multimapped reads (those that map to more than one transcript); and a total of 2,937 transcripts contained one or more predicted IEB. A total of 16,764 IEBs were predicted (on average 5.70 predicted IEB per transcript). These predicted IEBs were annotated and avoided during genotyping primer design.

A set of 96 SNPs was selected based on IEB prediction analysis for validation and genotyping on Fluidigm Genotyping System. From the 96 SNPs that were genotyped, 4 (4.17%) were categorized as *no signal*, while the remaining 92 SNPs were *polymorphic* with >80% call rate. Therefore, conversion and validation rates of 95.83% were achieved.

### Population genetics

**Fig 1:**
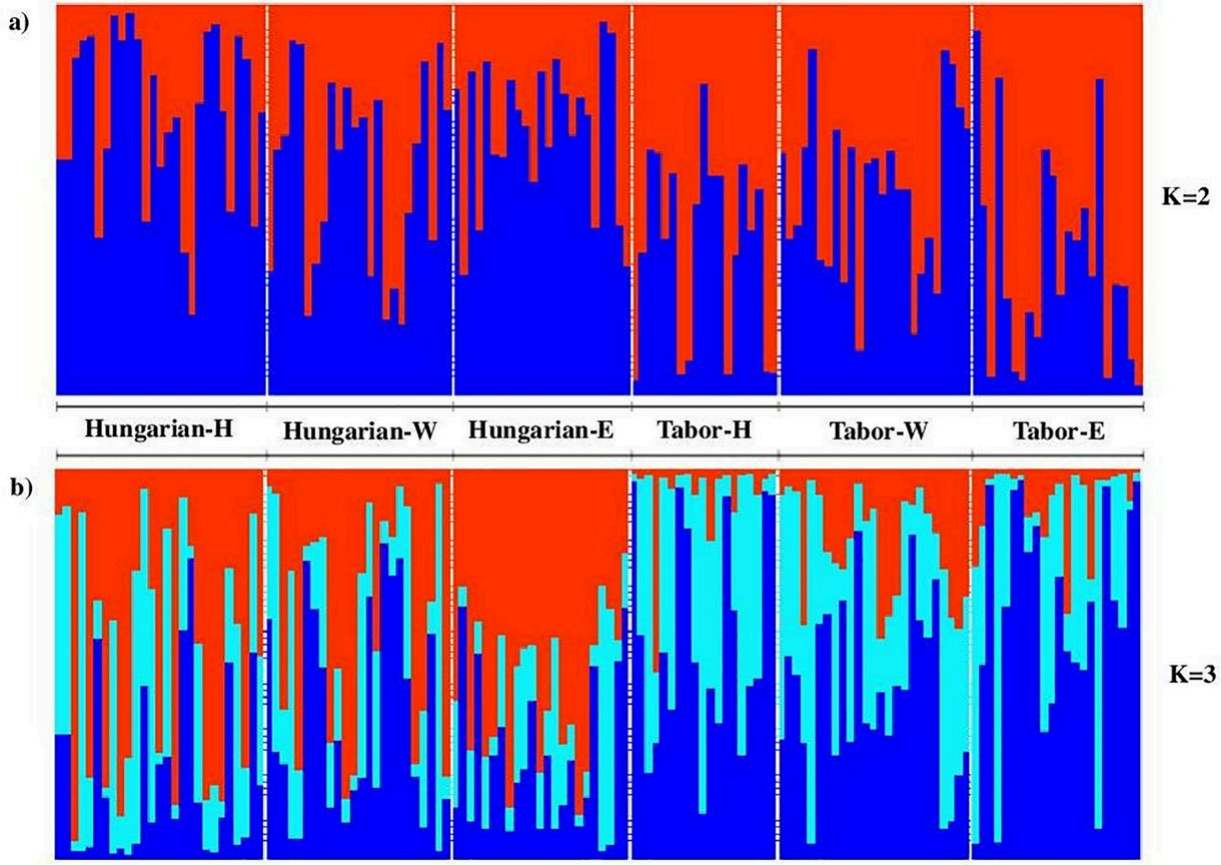
Results from Structure analysis for K = 2 (a) and K = 3 (b). Individuals corresponding to each breed (Hungarian, Tabor) and GH gene phylogroup genotype (H: Hybrid; W: Western; E: Eastern) are separated with vertical white bars.

Mean *H*_*o*_ and *H*_*e*_ for the Hungarian breed were 0.508 and 0.460, respectively. Similar levels of *H*_*o*_ (0.455) and *H*_*e*_ (0.458) were found in the Tabor breed. Tests of deviation from HWE for each locus revealed no significant departure from HWE after sequential Bonferroni correction. The Structure analysis evidenced population structure with K = 2 (Evanno method; Fig 1A), and K = 3 (Pritchard method; Fig 1B) being the most likely number of clusters. The average of the mean posterior probability (LnP(D)) estimated from 10 independent runs on K = 2 and K = 3 was -16533.7 and -16176.1, respectively. These clusters clearly indicate the differences between the two breeds, but not between the GH gene genotypes (Fig 1).

A total of six SNPs were detected as being under diversifying selection (positive alpha values); this is, they show extremely different allele frequencies in the two breeds. These outlier SNPs were located in the following genes: MRPL32 (39S ribosomal protein L32, mitochondrial), CENPF (Centromere Protein F), GRM1 (Glutamate Metabotropic Receptor 1), SPRY4 (Protein sprouty homolog 4), TRIP4 (Thyroid Hormone Receptor Interactor 4) and CN080 (Uncharacterized protein C14orf80 homolog) (Table 2). Of these six SNPs, all were found to be synonymous mutations, except interestingly for the SNP within *Activating signal cointegrator 1* (see Table 2) which encodes for either a Val (hydrophobic amino acid) or a Ser (polar amino acid). Since this substitutes a polar amino acid for a hydrophobic one, this SNP may lead to a change in protein function and should be further explored. Functional annotation revealed that most of these genes encoded proteins involved in transcription and translational regulation and structural organization of ribosome and mitochondria. Apart from these, the annotated gene Sprouty homolog 4-like was found to be involved in regulation of fibroblast growth and skeletal muscle fiber development, suggesting that the studied tench breeds might be adapted to different environments that affect growth related genes.

**Table 2:** Annotation of selected loci based top BLAST hit and GO ontology.

Locus ID	Genomic BLAST Hit	GO ID	e-value	Gene function
TR107177|c0_g1_i1	Sprouty homolog 4-like	GO:001602 GO:0021594GO:0030097GO:0040037GO:004874 GO:0070373	0.0E0	P: Negative regulation of fibroblast growth factor receptor signaling pathway; P: Rhombomere formation; P: Skeletal muscle fiber development; P: Hemopoiesis; P: Negative regulation of ERK1 and ERK2 cascade; C: Membrane
TR57930|c0_g1_i1	Centromere F	GO:0008134GO:0042803GO:0045502	0.0E0	F: Protein homodimerization activity; F: Transcription factor binding; F: Dynein binding
TR48380|c0_g1_i1	39S ribosomal L32, mitochondrial	GO:0005743GO:0005762GO:0003735GO:0016787GO:0006412	2.2 E-105	F: Structural constituent of ribosome; C: Mitochondrial large ribosomal subunit; C: Mitochondrial inner membrane; F: Hydrolase activity; P: Translation
TR71953|c0_g1_i1	Metabotropic glutamate receptor 1-like isoform X1	GO:0016020GO:0004871GO:0007165	1.1E-153	P: Signal transduction; C: Membrane; F: Signal transducer activity
TR96558|c0_g1_i2	Activating signal cointegrator 1	GO:0005634GO:0003713GO:0008270GO:0006366GO:0045893	0.0E0	C: Nucleus; F: Zinc ion binding; F: Transcription coactivator activity; P: Positive regulation of transcription, DNA-templated; P: Transcription from RNA polymerase II promoter
TR56671|c0_g1_i1	Uncharacterized protein C14orf80 homolog isoform X1	-	0.0E0	-

After removing the 6 outlier SNPs, a set composed of 86 SNP markers was used for studying neutral genetic differentiation and inbreeding. Pairwise F_ST_ estimates, within each breed, among E and W phylogroups and EW hybrid (H) were not significant; in contrast, all F_ST_ values were significant when pairwise comparisons between the two breeds were tested (Table 3). Overall, F_ST_ value between the two breeds was low but significant (F_ST_ = 0.0450, p-value <0.0001). Additionally, F_IS_ within each breed was not significant, indicating homogeneity within breeds. In summary, individuals within breeds show homogeneous allele frequencies without regard to GH gene genotype, whereas individuals of the two breeds (even if they both are a mixture of E and W phylogroup haplotypes) are genetically different. Genotyping results of all 92 SNPs markers have also been included in the S19 Table.

**Table 3:** Pairwise F_ST_ (below diagonal) and p-values (above diagonal) among tench breeds (Hungarian, Tabor) and GH gene phylogroups genotype (H: Hybrid; W: Western; E: Eastern).

	Hungarian -H	Hungarian -W	Hungarian -E	Tabor-H	Tabor-W	Tabor-E
**Hungarian-H**	-	0.2022	0.1592	0.0000	0.0000	0.0000
**Hungarian -W**	0.0012	-	0.0429	0.0379	0.0000	0.0000
**Hungarian -E**	0.0025	0.0083	-	0.0787	0.0504	0.0000
**Tabor-H**	0.0619*	0.0000	0.0000	-	0.1973	0.3936
**Tabor-W**	0.0399*	0.0274*	0.0000	0.00538	-	0.0049
**Tabor-E**	0.0579*	0.0318*	0.0687*	0.00150	0.0218	-

* significant value

## Discussion

The major challenge of transcriptome-derived SNPs is marker “drop-out” during the validation step; the most significant factor is if a SNP spans an IEB. For instance, 64% of genotyping failures have been reported in EST-derived SNPs in catfish due to the proximity of SNPs to IEB [65]. The most evident cause for such genotyping failure is the presence of priming site at SNPs loci leading to non-base pairing of primers or expected amplification product is too large for amplification due to presence of intron between priming sites. Therefore, the key for successful SNP validation is avoidance of IEBs. In this study, the approach devised by [66] and applied successfully to European anchovy [38] was used to avoid the problem related to IEBs. In this method, the assembled transcript sequences were aligned to genome sequences of tench to identify the IEB. By selecting the SNPs not spanning an IEB, we obtained the highest conversion and validation rates of transcriptome-derived SNPs obtained to date for a non-model species.

In this study, using the validated SNPs we have demonstrated that the two tench breeds show low but significant genetic differentiation, even with their similar genetic structure concerning their phylogroup based gene pool. The ancestral populations that formed the two tench phylogroups separated about 0.064 to 1.6 million years ago as revealed from 1.6% sequence divergence of cytochrome b mitochondrial gene [21]. The western (W) and Eastern (E) phylogroup significantly differs also in sequences of nuclear DNA e.g. the second intron of the actin gene, an intron of the gene coding for the ATP synthase β subunit, the first intron of the gene coding for the S7 ribosomal protein [21] and GH gene [6]. Due to the long history of tench phylogroup separation and individual evolution it is expected that the phylogroups would differ significantly also in physiological and biological functions resulting from nucleotide polymorphisms of functional genes. Therefore, our transcriptome-derived SNP array could be used for screening tench populations that still contain haplotypes of pure Western and pure Eastern phylogroup or F1 hybrid generation between pure W and E tench populations. Unfortunately, tench populations that bear pure Western haplotypes are very scarce or even absent [21] and we did not have such population in our collection. The Hungarian and Tabor breeds are, after several generations of mating fish with haplotypes of both phylogroups, a mosaic of both phylogroups due to free combination of chromosomes, crossing overs between homologous chromosomes and other possible processes that appear during formation of gametes. Based on F_ST_ values inferred from 86 SNPs it can be indirectly assumed that the SNPs genotypes were not significantly different for fish having Eastern, Western or hybrid GH gene genotype [22] within both Tabor and Hungarian breed. If the rate of phylogroup introgression within breeds were low, the degree of differentiation among fish displaying different GH gene genotype would be expected due to previously mentioned divergence between phylogroups in other genetic markers. On the other hand, significantly different F_ST_ values were observed between the two breeds with no matter to what GH gene genotype the fish belonged. The within-breed gene flow is corroborated by previous studies that show no negative fitness consequences derived from two phylogroup-mixed tench populations under cultured conditions [78]. In summary, six generations of within-breed isolated reproduction under cultured conditions allowed breed identity determination using the transcriptome-derived SNP array.

Moreover, apart from neutral levels of genetic differentiation, the SNPs in this study are transcriptome-derived markers and their variation in genes is informative for differential selection or adaptation in each breed. In this study, high allelic differentiation between both breeds was observed in growth-related genes, which might point to differential natural and human-affected selection, breeding and evolutionary history of Hungarian and Tabor tench breeds and/or stocks they were established from. Taking into account that the sequence of the GH (growth hormone) gene has 0.8% divergence in both tench phylogroups [6], we propose the following hypothesis: adaptive differences between breeds arise from differential composition of individuals from each phylogroup in each breed, giving to Hungarian and Tabor breeds different weight to their adaptation affecting growth related genes. However, further studies with protein sequencing of genes under selection are needed to corroborate the hypothesis presented here, as most of the SNPs found in the genes under selection have arisen due to synonymous mutations and will not lead to a change in the protein configuration. Insignificant association between GH gene genotype and SNP array also indicates that there is no linkage between our SNPs and the GH gene. However, this result does not say anything about association of these two markers to growth-related traits. It seems that effects of SNP array and GH gene genotype polymorphism on the growth-related traits will be (if any) independent of each other.

This study represents the first large-scale sequencing effort for SNP discovery and validation in tench. Although restriction-site associated DNA sequencing (RADseq) or double digest RADseq (ddRADseq) can generate large data set, SNPs derived from these approaches mostly fall into non-coding or unknown regions. Transcriptome derived SNPs are directly associated with functional regions in the genome and can give more information for 92 SNPs in coding region than hundreds or thousands of SNPs derived from non-coding or (not identified) regions. The validated SNPs can be used in further genetic studies for finding genes and/or DNA sequences associated with trait of importance.

## Conclusions

The SNP discovery approach followed in the present study was developed for transcriptome-derived SNP discovery in European anchovy [38], and Atlantic mackerel [43] with successful conversion and validation rates. This approach can be used to discover large number of transcriptome-derived SNPs in any non-model species. In addition, our approach identifies SNPs in the transcriptome: these SNPs can be annotated and in some cases, as evidenced here, they are under natural selection. We showed that the SNPs array in tench is strong enough to distinguish tench breeds and that it might be useful for studies focused on searching the range of associations between DNA sequence and traits of importance. Overall, it was verified that transcriptome-derived SNPs may informs us not only about neutral genetic differentiation and population genetic structure (e.g. [37, 39]), but also about the functional role of the differences observed between populations or ecotypes

## Supporting information

S1 Fig GenomeScope profile. (TIFF)

S2 Fig Transcript-length distribution. (TIF)

S3 Fig Gene—Transcript distribution after TransDecoder prediction. (TIF)

S4 Fig Blastp hits distribution by organism. Organism is encoded by 5 letters (ASTMX *Astyanax mexicanus*, DANRE *Danio rerio*; ICTPU *Ictalurus punctatus*; LEPOC; ONCMY *Oncorryncus mykiss*; ORENI *Oreochromis niloticus*; POEFO; TAKRU *Takifugu rubripes*; TETNG; XIPMA).
(TIF)

S5 Fig Blastx hits distribution by organism. Organism is encoded by 5 letters (ASTMX *Astyanax mexicanus*, DANRE *Danio rerio*; ICTPU *Ictalurus punctatus*; LEPOC; ONCMY *Oncorryncus mykiss*; ORENI *Oreochromis niloticus*; POEFO; TAKRU *Takifugu rubripes*; TETNG; XIPMA).
(TIF)

S6 Fig Frequency distribution of Pfam domains (top 20). (TIF)

S7 Fig EggNOG distribution by ID (top 20). (TIF)

S8 Fig Gene Ontology summary. (TIF)

S9 Fig Joint GO level 2 distribution (top 30). (TIF)

S10 Fig Distribution of the Biological Process Gene Ontology terms. (TIF)

S11 Fig Distribution of the Cellular Component Gene Ontology terms. (TIF)

S12 Fig Distribution of the Molecular Function Gene Ontology terms. (TIF)

S1 Table Details of samples included in transcriptome sequencing. (XLSX)

S2 Table Details of samples included in genome sequencing. (XLSX)

S3 Table Transcriptome and genome sequencing results. (XLSX)

S4 Table Genome size estimation. (XLSX)

S5 Table Results obtained from the trimming performed in order to do transcriptome assembly. (XLSX)

S6 Table Backmapping of the reads. (XLSX)

S7 Table BUSCO. (XLSX)

S8 Table UniRef90 results from proteome querying (blastp). (XLSX)

S9 Table UniRef90 results from transcriptome querying (blastx). (XLSX)

S10 Table Summary of blastp results against UniRef90 by organism. Organism is encoded by 5 letters (ASTMX *Astyanax mexicanus*; DANRE *Danio rerio*; ICTPU *Ictalurus punctatus*; LEPOC; ONCMY *Oncorryncus mykiss*; ORENI *Oreochromis niloticus*; POEFO; TAKRU *Takifugu rubripes*; TETNG; XIPMA).
(XLSX)

S11 Table Pfam hits. (XLSX)

S12 Table Pfam hits summarized by domain. (XLSX)

S13 Table EggNOG results. (XLSX)

S14 Table Summary of Gene Ontology results (level 1). (XLSX)

S15 Table Gene Ontology annotation of the transcriptome and proteome. (XLSX)

S16 Table Trimming for SNP discovery (DNA). (XLSX)

S17 Table Trimming for SNP discovery (RNA). (XLSX)

S18 Table Mapping. (XLSX)

S19 Table Genotyping results of all the 92 SNPs markers. (XLSX)
