# Supplementary figures and images for: A novel transcriptome-derived SNPs array for tench (Tinca tinca L.)
Source: PLoS One. 2019 Mar 19;14(3):e0213992. doi: 10.1371/journal.pone.0213992 (PMC6424483; doi:10.1371/journal.pone.0213992)

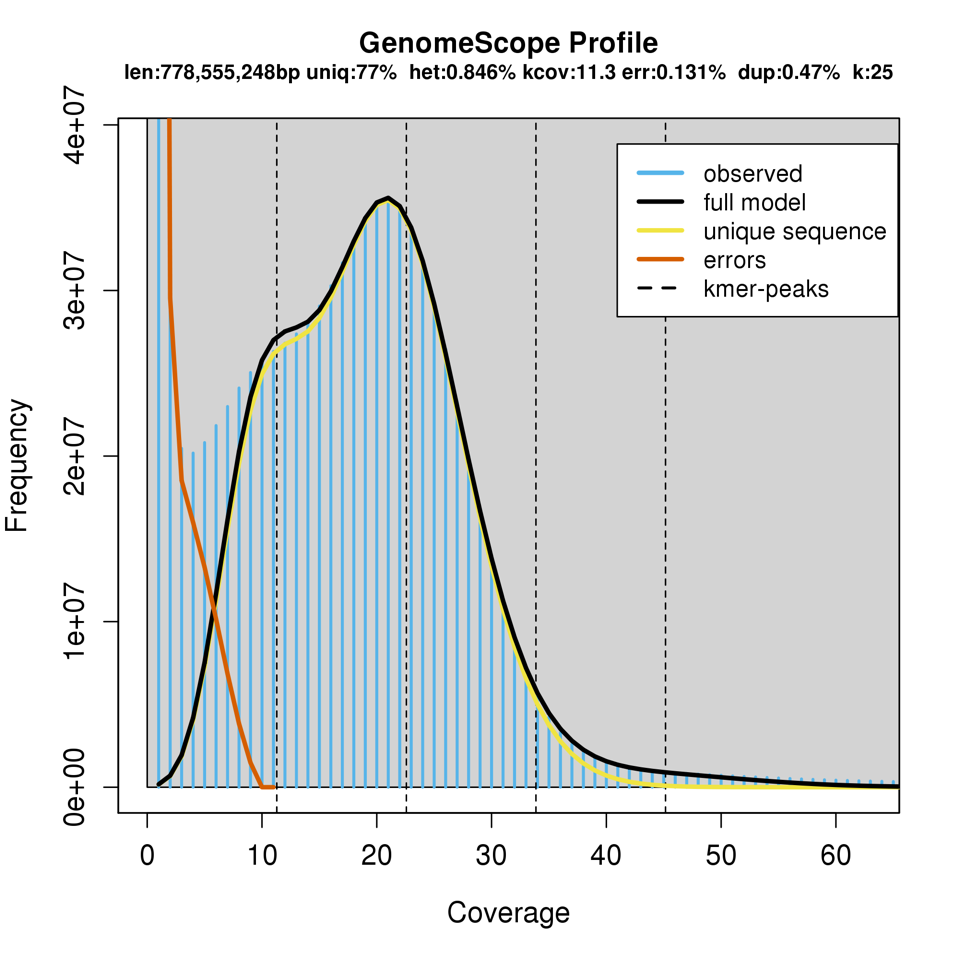

Supplement: S1 Fig — (TIFF) [file pone.0213992.s001.tiff]

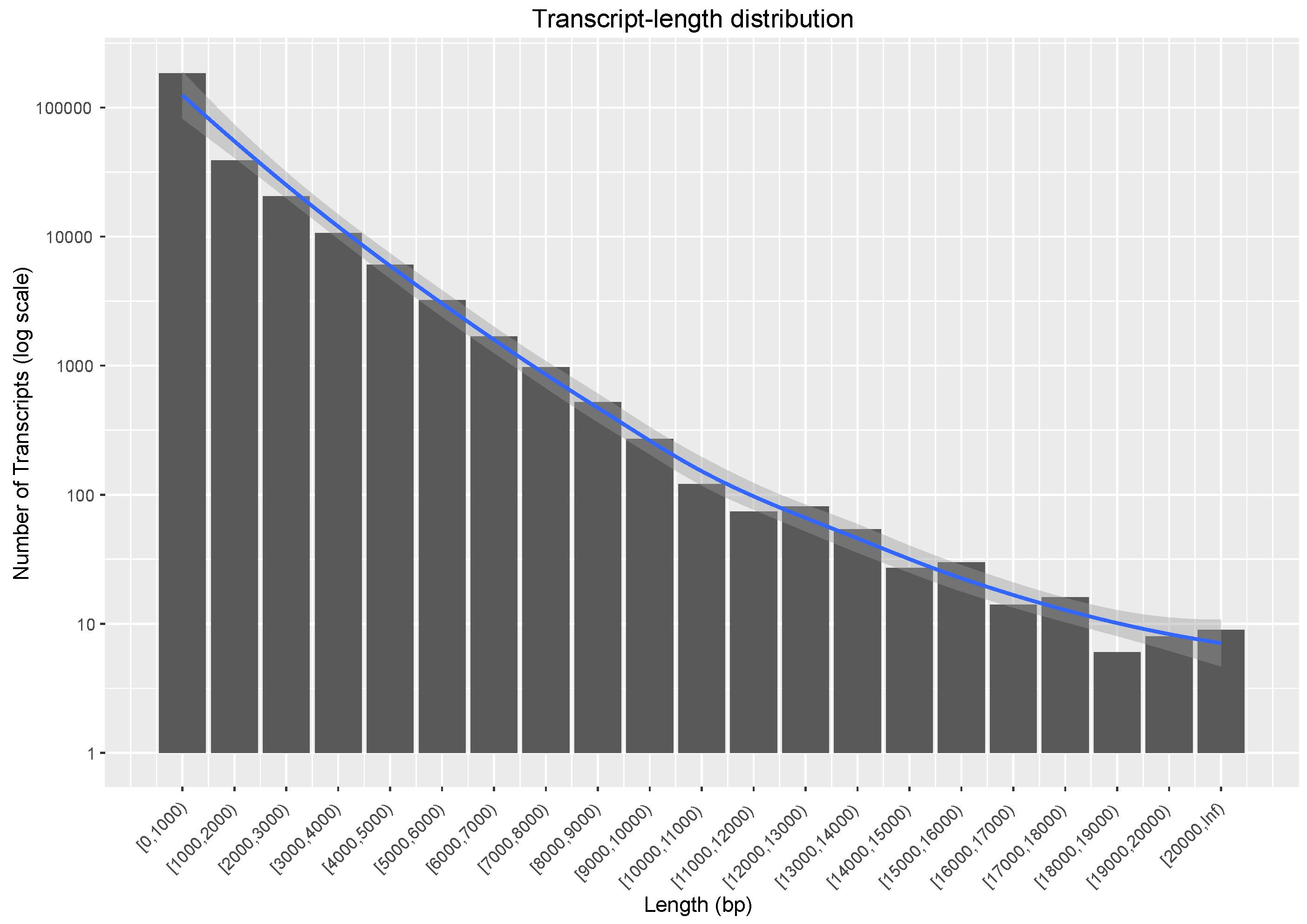

Supplement: S2 Fig — (TIF) [file pone.0213992.s002.tif]

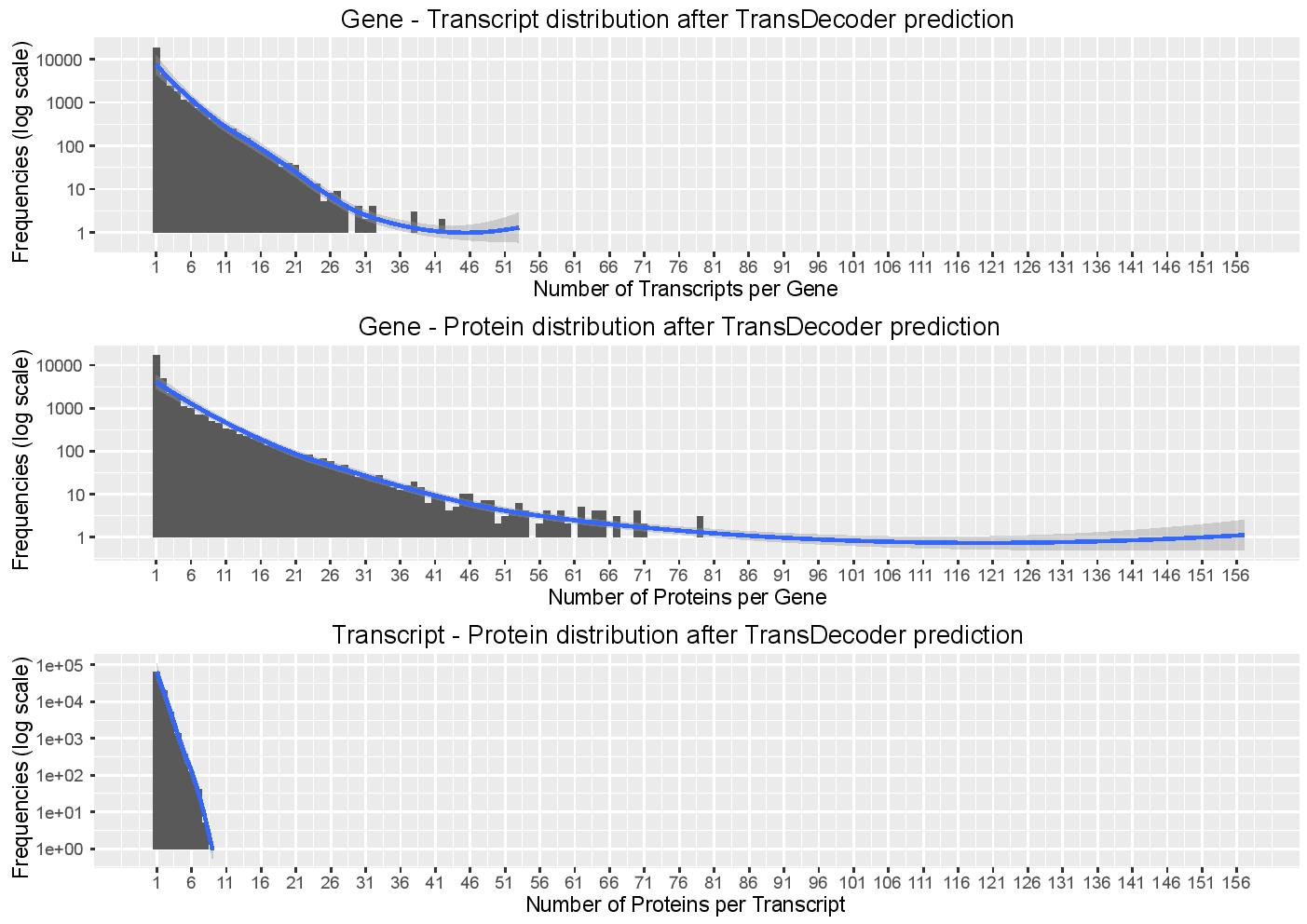

Supplement: S3 Fig — (TIF) [file pone.0213992.s003.tif]

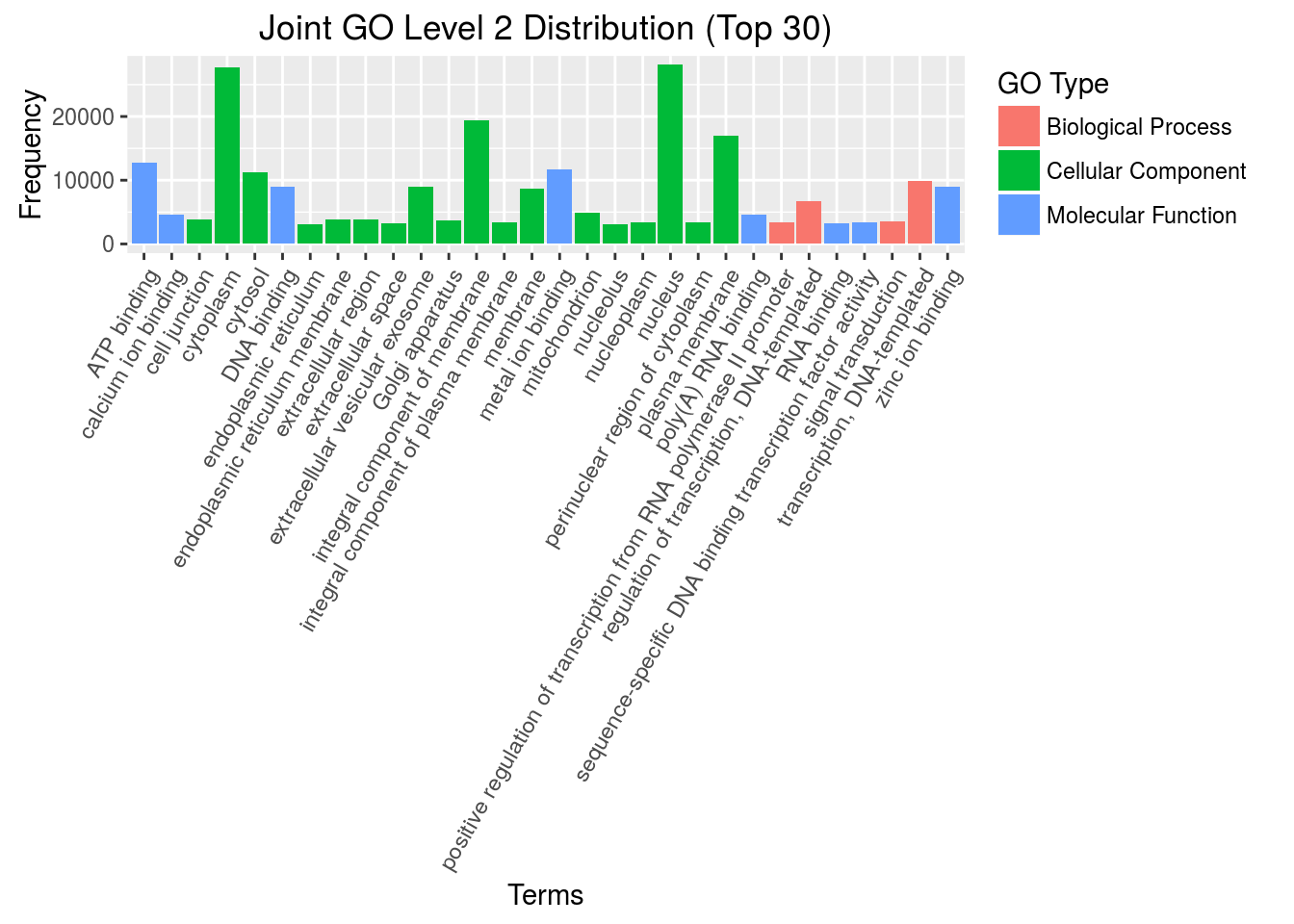

Supplement: S9 Fig — (TIF) [file pone.0213992.s009.tif]
